# Crimean-Congo Hemorrhagic Fever Infection in the Small Ruminant Population in N. Macedonia: A Seroepidemiological Study as a Step Towards Better Understanding of the CCHF Epidemiology in the Country

**DOI:** 10.3390/pathogens15060637

**Published:** 2026-06-16

**Authors:** Ivan Matevski, Zagorka Popova Hristovska, Igor Djadjovski, Kiril Krstevski

**Affiliations:** Faculty of Veterinary Medicine—Skopje, Ss. Cyril and Methodius University in Skopje, 1000 Skopje, North Macedonia; zaga@fvm.ukim.edu.mk (Z.P.H.); igordz@fvm.ukim.edu.mk (I.D.)

**Keywords:** Crimean-Congo hemorrhagic fever, CCHFV, small ruminants, seroprevalence, tick-borne zoonoses, One Health

## Abstract

Crimean-Congo hemorrhagic fever (CCHF) is a tick-borne zoonotic disease of significant public health concern, particularly in endemic regions. However, data on the distribution and circulation of Crimean-Congo hemorrhagic fever virus (CCHFV) in animal populations remain limited, despite their importance for assessing virus circulation and infection patterns. A cross-sectional seroepidemiological study was conducted in North Macedonia (N. Macedonia) to determine the seroprevalence rates of CCHFV in small ruminants and to identify areas at increased risk of virus circulation. A total of 1992 sera samples from sheep and goats were tested for Crimean-Congo hemorrhagic fever virus (CCHFV) antibody using a commercial enzyme-linked immunosorbent assay (ELISA). Data on species, age, animal origin, and risk factors (questionnaire) were collected and analyzed. Overall, true seroprevalence, calculated by adjusting the apparent seroprevalence according to the diagnostic performance of the ELISA assay in order to estimate the actual prevalence in the studied population, was 25.02% (493/1992; 95% CI 23.11–26.94). Sheep showed higher seroprevalence rates, 27.34% (397/1452; 95% CI 25.11–29.69), compared to goats, 17.78% (96/540; 95% CI 14.78–21.23). Seroprevalence rates varied markedly across regions, ranging from 2.41% to 49.80%, with the highest values observed in the Eastern and Vardar regions. Seroprevalence rates increased with age, reaching the highest values in animals aged ≥ 5 years, 27.27% (81/297; 95% CI 22.52–32.60). Small ruminants, particularly sheep, may serve as useful indicators for defining the high-risk areas for CCHFV transmission, providing valuable support for a One Health approach.

## 1. Introduction

Crimean-Congo hemorrhagic fever (CCHF) is a tick-borne, fatal viral zoonotic disease caused by Crimean-Congo hemorrhagic fever virus (CCHFV) [1]. The virus belongs to the genus *Orthonairovirus*, family *Nairoviridae*, order *Bunyavirales* [1,2]. The viral genome is segmented and comprises tripartite negative-sense, single-stranded ribonucleic acid (S, M, and L segments) [3]. Global trends such as climate change, intensive trade and travel, political instability, and insurgencies affect the epidemiology of many diseases and place many vector-borne zoonotic diseases high on the priorities of public health authorities [4]. The importance of CCHF has increased in the last few decades due to its epidemic potential and its bioterroristic risk to public health. Furthermore, the disease was included by the World Health Organization (WHO) in the list of important emerging infectious diseases with pandemic potential [5,6]. According to the European Food Safety Authority (EFSA), CCHF has been identified as a priority zoonotic disease for integrated and coordinated One Health surveillance in Europe due to its zoonotic and transboundary nature, vector-borne transmission, expanding geographic risk, and the need for early detection and response mechanisms [7].

The ecological cycle of CCHFV is broad and contains transstadial, transovarial, and venereal transmission among the tick population, and tick co-feeding. Various vertebrates, like sheep, goats, cattle, horses, pigs, wild animals, birds, and humans, are included in this tick-vertebrate-tick cycle [8,9,10]. Ticks, especially of the genus *Hyalomma*, play an essential role in the amplification and spread of the virus as a viral vector and reservoir [8]. Bites from infected ticks remain the main route of transmission of CCHFV in humans and animals [8,11]. CCHFV infection in livestock is generally asymptomatic or subclinical, and during the viremia, it acts as a reservoir of CCHFV for other participants in the natural cycle of the virus (uninfected ticks, humans, and other animals) [12,13,14]. Following transient viremia, livestock seroconvert and develop detectable specific anti-CCHFV antibodies [15].

The primary tools for assessing the prevalence of CCHFV infection are serological diagnostic techniques. Enzyme-linked immunosorbent assays (ELISAs), especially IgG ELISA-based assays, are widely used in seroepidemiological studies. Conversely, immunofluorescence and virus neutralization assays may provide confirmatory support, taking into account differences in diagnostic performance and possible cross-reactivity [16,17]. Serological screening of domestic animals, especially populations with prolonged exposure to tick bites (e.g., small ruminants grazing in open pastures), seems to be an appropriate and feasible approach for epidemiological investigations and disease tracking [18]. The presence or absence of antibodies against the virus in small ruminants is a good indicator of the circulation of the CCHFV in a specific territory [18,19]. This forms the basis for serological screening and surveillance, early geographic detection of virus circulation, and outbreak risk assessment [19,20].

CCHFV has a wide geographic distribution and is endemic in more than 30 countries across Africa, Asia, the Middle East, and Europe, with Southeast Europe, particularly the Balkan Peninsula, constituting an important region for endemic circulation and re-emergence [19,20,21,22,23,24,25]. Within this regional context, in N. Macedonia, six human CCHF cases have been officially confirmed so far. The first one was identified in the 1970s. More than 50 years later, five cases were laboratory-proven in 2023 and 2024, one of which was fatal [6,26,27]. A systematic surveillance of the seroepidemiological situation in animals in N. Macedonia has not been carried out yet. The available data are limited, and they were obtained in the framework of projects whose primary aim was the validation of new diagnostic methods [18,19]. In addition, serological evidence in animals has recently been documented within a One Health investigation [28].

Given the lack of systematically organized serosurveillance data, this study aims to assess the current seroepidemiological situation of CCHF in N. Macedonia through testing of the presence of antibodies in small ruminants as proven indicators to evaluate the prevalence of the CCHFV infection, define possible regional preferences and risk areas, and evaluate a possible association of specific factors with the disease.

## 2. Materials and Methods

### 2.1. Study Area and Sampling Design

The study was carried out in N. Macedonia, a landlocked, agriculture-based country, covering 25,713 square kilometers, situated in Southeastern Europe between latitudes 40°50′ and 42°20′ N, and and longitudes 20°27′30″ and 23°05′ E [29]. The sera were sampled across the whole territory, with each of the eight NUTS3-level regions representing separate epidemiological units [30].

The study is a cross-sectional study with simple random sampling. Archived sera from small ruminants, including sheep (*Ovis aries*) and goats (*Capra hircus*), were collected during March–December 2023 within the framework of the national program for the control and eradication of brucellosis in sheep and goats. Blood samples were obtained by jugular venipuncture, and sera were separated by centrifugation and transferred into sterile tubes. All samples were stored at −20 °C until testing.

**Figure 1:**
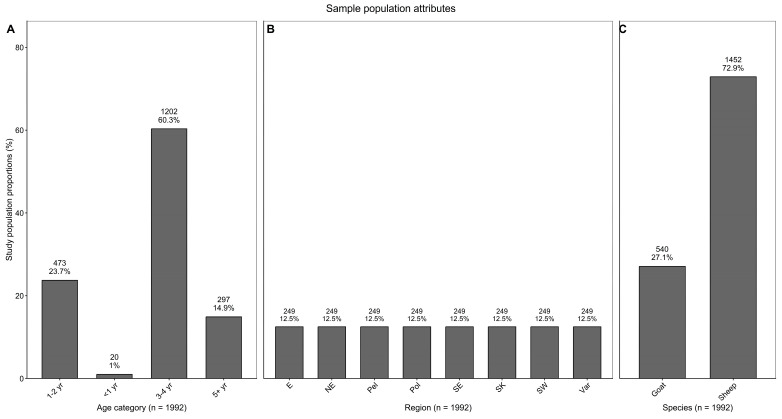
Sample population attributes. (**A**) Distribution of sampled animals by age category. (**B**) Distribution of sampled animals by statistical region. (**C**) Distribution of sampled animals by species. Bars indicate the proportion (%) of the study population, with the number of animals and corresponding percentage shown above each bar.

The sample size was estimated using Epitools (AusVet, Fremantle, Australia, Available online: http://epitools.ausvet.com.au/ (accessed on 5 May 2024)) [31]. The calculation assumed an expected prevalence of 20%, test sensitivity of 99%, and specificity of 100%, a desired precision of 5%, and a 95% confidence level. This approach ensured that the calculated sample size was representative of the actual prevalence in the target population. According to the estimation, a total sample size of 1992 sera samples originating from 295 herds was determined using those parameters (Figure 1).

### 2.2. Enzyme-Linked Immunosorbent Assay (ELISA)

Sheep and goat serum samples were plated on microtiter plates according to a previously prepared sequence. Plated sera were then inactivated by heating at 58 °C for 30 min to minimize unspecific binding. After inactivation, sera samples were processed according to the manufacturer’s protocol of the ID Screen CCHF Double Antigen Multi-species ELISA kit (IDvet, Grabels, France) [32]. The ELISA kit has been validated for the detection of antibodies against CCHFV in cattle, sheep, goats, and other suspected species [19] and is based on the recombinant N-protein of the IbAr10200 virus [18,32]. According to the manufacturer and validation report, the ELISA has a sensitivity of 98.9% and a specificity of 100%, with no cross-reactivity observed in validation studies using related nairoviruses, including Dugbe virus, Hazara virus, and Nairobi Sheep Diseases Virus. However, due to the observed antigenic similarity, cross-reactivity with Aigai virus cannot be ruled out.

The results were interpreted according to the manufacturer’s instructions using the sample-to-positive percentage (S/P%), calculated as S/P% = (ODsample/OD_PC_) × 100. Samples with S/P values ≤ 30% were considered negative, whereas samples with S/P values > 30% were considered positive.

For quality control purposes, test performance was continuously monitored by the inclusion of one internal positive control serum (out of the kit control) and by calculating inter-assay variability. Additionally, intra-assay variability was evaluated by testing a separate internal positive control serum as a replicate in a single test run. Internal positive control sera were obtained from two sheep previously tested with a strong ELISA signal (Supplementary Table S1). This design ensured a robust evaluation of the test performances in accordance with internationally recognized criteria for diagnostic test validation [17].

### 2.3. Questionnaire Design and Administration

A structured questionnaire was developed to collect herd-level information on potential risk factors associated with exposure to tick-borne infections. The questionnaire included variables on terrain type, herd size, herd mixing practices, introduction of new animals, movement patterns, tick exposure, and tick control measures.

The questionnaire was implemented using the online version of the KoboToolbox platform (KoboToolbox, Cambridge, MA, USA), available at https://www.kobotoolbox.org/ (accessed on 19 February 2025) and administered in the local language through telephone interviews with herd owners. Questionnaire data were analyzed descriptively to summarize herd-level characteristics.

### 2.4. Statistical Analysis

The serological results were binarized as either positive or negative based on the optical density (OD) values according to the manufacturer’s protocol. Seroprevalence was calculated as the proportion of seropositive animals and expressed as a percentage with corresponding 95% confidence intervals (95% CIs) estimated using binomial methods. Apparent seroprevalence was defined as the observed proportion of ELISA-positive animals among the total number of tested animals. True (or actual) prevalence was calculated using the Rogan-Gladen correction by adjusting the calculated apparent seroprevalence for the diagnostic performance of the ELISA test (sensitivity = 0.989; specificity = 1.00). Subgroup-specific estimates are presented as apparent seroprevalence.

Factors associated with seropositivity were assessed using a Bayesian mixed-effects logistic regression model, including region, age category, and species as fixed effects and herd as a random intercept to account for clustering of animals within herds. The results are presented as odds ratios (ORs) with 95% CI, and herd-level clustering was quantified using the intra-class correlation coefficient (ICC).

A standard logistic regression model without random effects was fitted as a sensitivity analysis. Post hoc pairwise comparisons were performed using estimated marginal means with Tukey adjustment.

Statistical significance was set at *p* < 0.05, and highly significant results were reported as *p* < 0.001. All analyses were performed using R software (version 4.5.3).

## 3. Results

### 3.1. Characteristics of the Study Population

The study population comprised 1992 small ruminants originating from 295 herds and distributed across eight statistical regions. An equal number of samples was collected from each region. Sheep accounted for a higher proportion of the sampled population, corresponding to the actual proportion of sheep and goats in each of the regions. Animals in the 3–4 yr age category represented the largest proportion of the study population. The distribution of the study population according to species, age categories, and regions is presented in Figure 1.

### 3.2. Overall Seroprevalence

**Figure 2:**
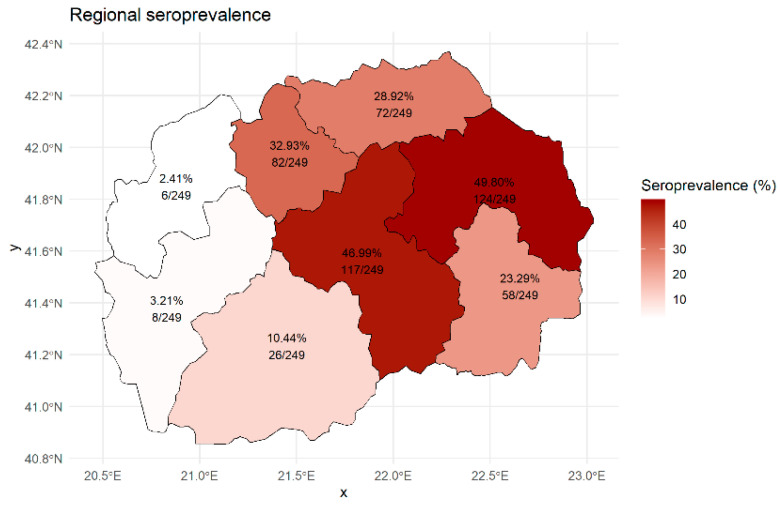
Regional seroprevalence of CCHFV antibodies by statistical region (epi unit). Values indicate apparent seroprevalence (%) and the number of positive animals among tested animals. The figure was generated in R software (version 4.5.3) using publicly available NUTS level 3 geographical boundary data obtained from the Eurostat/GISCO database.

A total of 1992 sera samples were tested, and the overall apparent seroprevalence was 24.75% (493/1992; 95% CI 22.9–26.69), while the estimated true prevalence was 25.02% (493/1992; 95% CI 23.11–26.94). Seroprevalence varied across regions, with the highest values observed in the Eastern region, 49.80% (124/249; 95% CI 43.64–55.97), and the lowest in the Polog region, 2.41% (6/249; 95% CI 1.11–5.16) (Figure 2).

Among tested species, the highest seroprevalence was found in sheep, 27.34% (397/1452; 95% CI 25.11–29.69), whereas goats showed a lower seroprevalence, 17.78% (96/540; 95% CI 14.78–21.23). Across age categories, seroprevalence increased with age, with higher values observed in the age category ≥ 5 years, 27.27% (81/297; 95% CI 22.52–32.60), and decreased with decreasing age, reaching the lowest found in <1 year, 0.00% (0/20; 95% CI 0.00–16.11), as shown in Table 1 (Supplementary Figures S1 and S2).

### 3.3. Factors Associated with Seropositivity—Bayesian Mixed-Effects Logistic Regression Analysis

**Figure 3:**
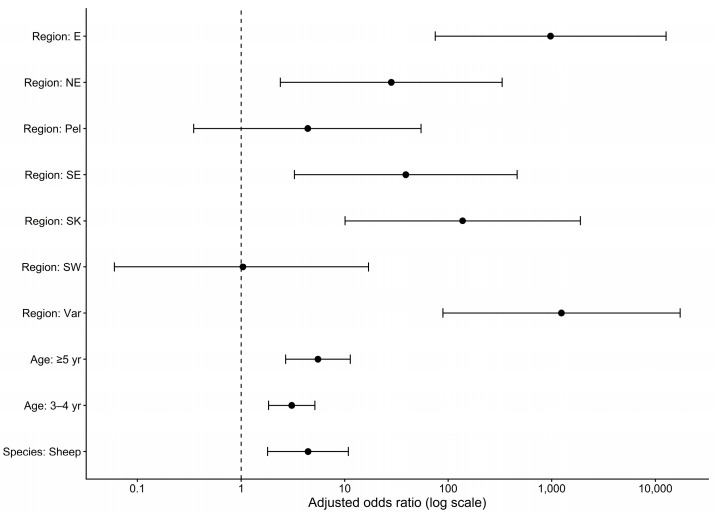
Forest plot of adjusted odds ratios (aORs) for factors associated with CCHFV seropositivity in small ruminants, estimated using the BLME model. Points represent adjusted odds ratios, and horizontal lines represent 95% confidence intervals (CIs). The vertical reference line at an odds ratio of 1 indicates no association.

Age, region, and species were significantly associated with seropositivity (Table 2; Figure 3). Higher odds of seropositivity were observed in animals belonging to the ≥5 years age group compared to the reference age category (OR = 5.52, 95% CI 2.69–11.33, *p* < 0.001). Compared to the reference Polog region, animals from the Vardar (OR = 1242.07, 95% CI 88.99–17,337.13, *p* < 0.001), Eastern (OR = 974.88, 95% CI 75.00–12,671.87, *p* < 0.001), Skopje (OR = 137.92, 95% CI 10.09–1884.75, *p* < 0.001), Southeastern (OR = 38.96% CI 3.27–463.64, *p* < 0.004), and Northeastern region (OR = 28.21, 95% CI 2.39–332.41, *p* < 0.008) had higher odds of seropositivity. No statistically significant difference was observed for Pelagonia and the Southwestern region compared to the reference region, as shown in Table 2 and Figure 3.

The intra-class correlation coefficient indicated a very high degree of clustering at the herd level (ICC = 0.85), supporting the inclusion of a random effect. Consistent with this, model fit statistics demonstrated that the BLME model provides a better fit to the data compared to the standard logistic regression model (Supplementary Tables S2 and S3).

Post hoc pairwise comparisons revealed significant differences between selected age categories, particularly between 1–2 years and ≥5 yr, and between 1–2 years and 3–4 years (*p* < 0.001). A significant difference was also observed between species (*p* < 0.001). Detailed results are presented in Supplementary Table S4.

No statistically significant associations were identified between questionnaire-derived and seropositivity in the mixed-effects analysis (Supplementary Table S5).

### 3.4. Descriptive Analysis of Questionnaire-Derived Risk Factors

The distribution of herd-level management and environmental variables is presented in Table 3. The majority of herds were located in hilly terrain (48.00%), while the lowest proportion was accounted for (22.67%). Most of the herds were classified as large herd size (56.00%), whereas medium herd size represents the least frequent category (21.33%). Herd mixing was reported in (8%) of herds, and the introduction of new animals was observed in (8%). Tick exposure varied across herds, with the low tick exposure being the most frequently reported (56.00%), while the high tick exposure accounted for the lowest proportion (5.33). Tick control measures were applied in (69.33%) of the herds.

### 3.5. Quality Control—Performance Verification of the ELISA Assay

Repeatability (intra-assay) and reproducibility (inter-assay) were evaluated by calculating the coefficient of variation (CV). The coefficient of variation (CV) for repeatability was 6.1%, while reproducibility showed a CV of 12.4%. Both values were within acceptable limits according to international standards, as shown in Table 4 [17].

## 4. Discussion

Crimean-Congo hemorrhagic fever is a vector-borne viral zoonotic disease with a wide geographic distribution [19,20,21,22,23,24,25]. It occurs mostly in Africa, Southeastern Europe, Asia, and the Middle East. It is a serious public health threat, especially for people with occupational exposure, including farmers, abattoir workers, and healthcare staff [14]. Effective control of zoonotic diseases relies on the One Health approach, ensuring coordination across animal, human, agricultural, and environmental sectors, along with continuous surveillance to identify foci, reservoirs, and transmission routes, and guiding prevention and control strategies [33,34]. Small ruminants act as a key reservoir for the virus, supporting virus maintenance and tick-borne transmission [35]. Seroepidemiological studies of CCHFV in small ruminants indicate viral circulation in certain territories and also help to identify the risk areas [19].

This study provides the first systematic surveillance of the seroepidemiologic situation in small ruminants in N. Macedonia. The results revealed a true seroprevalence of antibodies against CCHFV of 25.02% (493/1992; 95% CI 23.11–26.94) in small ruminants. Indistinguishable prevalence rates have been reported in Bulgaria (7.7% in sheep, 22.7% in goats), Kosovo (23.1%), and Albania (23%), while a significantly lower prevalence was observed in Greece (5%). These reports also support the evidence of widespread CCHFV circulation in neighboring countries [36,37,38,39,40]. Therefore, these findings provide a baseline for integrating periodic CCHFV serological monitoring of small ruminants into national disease control and prevention activities, supporting the timely identification of areas with increased virus circulation within a One Health framework.

Considerable regional differences in the prevalence of anti-CCHFV antibodies were observed, indicating heterogeneous exposure to the virus across the study area. Higher seroprevalence in regions such as Eastern, Vardar, Skopje, Northeastern, and Southeastern suggests increased levels of viral circulation compared to other regions. Our findings confirm marked regional variation in CCHFV seroprevalence consistent with reports from other endemic settings. A combination of ecological and epidemiological factors likely drives such differences. Interestingly, the four regions with the highest adjusted OR (Easter, Vardar, Skopje, and Northeastern regions) actually correspond to geographic areas with the most optimal topographic, climatic, and ecological characteristics that favor maintenance of more abundant tick populations. These regions are, for the most part, areas with long, hot summers, mild and dry winters, and open agricultural and pasture habitats. Therefore, a plausible hypothesis would be that this might explain the observed effect of the region on ORs, and this is definitely one that needs further in-depth study. Indeed, variation in the distribution and abundance of ticks, especially *Hyalomma* spp. as the principal vectors, together with climatic and environmental conditions, plays a central role in shaping local transmission dynamics of CCHFV [41]. In addition, the presence of suitable domestic and wild hosts, as well as livestock management practices, including grazing systems and animal movement, may further influence local exposure risk and contribute to the observed regional variation [41,42,43,44]. Such a wide variety of potential factors might influence the observed wide CI95% intervals and suggest the need for additional and more focused research.

At the age category level, higher seroprevalence was observed in animals ≥ 5 years of age, suggesting increased cumulative exposure to infected ticks over time. This pattern is consistent with previous studies reporting higher CCHFV seroprevalence in older livestock, which is commonly attributed to prolonged exposure to tick activity and repeated opportunities for infection [45,46]. For instance, a study in Senegal observed a seroprevalence of 16.2% in adult small ruminants, significantly higher than the 4.9% in younger animals [47].

Small ruminants play an important role in maintaining the tick population by providing blood meals that support the life cycle of the vector and facilitate virus transmission [44]. In the present study, sheep showed higher seroprevalence compared to goats, suggesting increased exposure to tick infestation. These interspecies differences are most likely associated with distinct grazing behaviors and feeding strategies. Sheep are primarily grazers, feeding close to the ground, whereas goats exhibit more flexible feeding behaviour, combining grazing and browsing [44,48]. As a result, sheep are more likely to come into contact with ticks inhabiting grasses and low-lying vegetation, which may increase their risk of exposure to CCHFV [49].

No statistically significant associations were observed between the questionnaire-derived herd-level risk factors and CCHFV seropositivity. This may partly reflect limited statistical power, as well as the complex epidemiology of CCHFV transmission, where multiple interacting ecological and epidemiological factors influence exposure risk [44,50].

When considered together with clinical and laboratory-confirmed human CCHF cases, the present findings gain additional epidemiological relevance. Five human cases were confirmed in 2023 and 2024, four of which occurred in rural areas across Skopje, Eastern, Northeastern, and Vardar regions, while one case was associated with nosocomial transmission. Notably, these regions correspond to those with the highest seroprevalence in small ruminants identified in this study [6,26,27,28]. This spatial concordance suggests that areas with increased seroprevalence in small ruminants may reflect increased local circulation of CCHFV and a higher risk of human exposure. These findings are consistent with previous studies indicating that seroprevalence in small ruminants may serve as a useful indicator of virus circulation in endemic settings [19,51].

In parallel with the seroepidemiological findings, recent investigations have provided baseline data on the diversity, distribution, and host associations of ixodid ticks in N. Macedonia. These data offer important insights into the structure of local tick populations and the presence of species of recognized epidemiological importance [52].

Building on these findings, future research should focus on the direct detection of CCHFV in tick populations, along with molecular and genetic characterization, to better define the role of different tick species in virus circulation and to improve understanding of the regional and global epidemiology of CCHFV. In addition, surveillance should be expanded to include other tick-borne pathogens of regional relevance, such as *Anaplasma* spp., *Babesia* spp., *Rickettsia* spp., and tick-borne encephalitis virus (TBEV), which have been reported in parts of the Balkan region [53,54,55].

## 5. Conclusions

This study represents the first nationwide assessment of CCHFV exposure in small ruminants. The findings indicate that small ruminants, particularly sheep, may serve as useful indicators for identifying areas at increased risk of CCHFV transmission. The observed correspondence between regions with higher seroprevalence and reported human cases further validates the relevance of this conclusion.

These results highlight the value of animal-based surveillance for identifying high-risk areas and reinforce the importance of a One Health approach in understanding and managing the disease.

## Figures and Tables

**Table 1:** Seroprevalence of CCHFV antibodies in different categories (species, age, and region).

Variable	Tested (*n*)	Positive (*n*)	Prevalence	95% CI (%)
Species: Sheep	1452	397	27.34%	25.11–29.69
Species: Goat	540	96	17.78%	14.78–21.23
Age: <1 yr	20	0	0.00%	0.00–16.11
Age: 1–2 yr	473	85	17.97%	14.77–21.68
Age: 3–4 yr	1202	327	27.20%	24.76–29.79
Age: ≥5 yr	297	81	27.27%	22.52–32.60
Region: Eastern	249	124	49.80%	43.64–55.97
Region: Northeastern	249	72	28.92%	23.64–34.83
Region: Pelagonia	249	26	10.44%	7.23–14.86
Region: Polog	249	6	2.41%	1.11–5.16
Region: Skopje	249	82	32.93%	27.39–38.99
Region: Southeastern	249	58	23.29%	18.47–28.93
Region: Southwestern	249	8	3.21%	1.64–6.21
Region: Vardar	249	117	46.99%	40.88–53.19

**Table 2:** Bayesian mixed-effects logistic regression analysis of factors associated with seropositivity.

Predictor	Adjusted OR	95% CI	*p*-Value
Eastern	974.88	75.00–12,671.87	<0.001 *
Northeastern	28.21	2.39–332.41	0.008 *
Pelagonia	4.40	0.35–54.69	0.249
Southeastern	38.96	3.27–463.64	0.004 *
Skopje	137.92	10.09–1884.75	<0.001 *
Southwestern	1.04	0.06–16.99	0.980
Vardar	1242.07	88.99–17,337.13	<0.001 *
<1 yr	0.00	0.00–58.0	0.931
≥5 yr	5.52	2.69–11.33	<0.001 *
3–4 yr	3.08	1.84–5.16	<0.001 *
Sheep	4.43	1.80–10.86	0.001 *

* Statistically significant difference at *p* ≤ 0.05, OR = odds ratio, CI = confidence interval. Heard was included as a random intercept. Reference categories: region = Polog, age = 1–2 yr, species = goat.

**Table 3:** Distribution of questionnaire-derived herd-level variables related to management practices and tick exposure.

Variable	Category	No. of Herds	Percent (%)
Terrain	Hilly	36	48.00
	Lowland	22	29.33
	Mountainous	17	22.67
Herd size	Large	42	56.00
	Medium	16	21.33
	Small	17	22.67
Herd mixing	No	69	92.00
	Yes	6	8.00
New animals	No	65	86.67
	Yes	10	13.33
Movement range	Local	49	65.33
	No Movement	2	2.67
	Outside Municipality	3	4.00
	Within Municipality	21	28.00
Tick exposure	High	4	5.33
	Low	42	56.00
	Moderate	17	22.67
	No Ticks	12	16.00
Tick control	No	23	30.67
	Yes	52	69.33

**Table 4:** Quality control results for the CCHFV ELISA.

Parameter	Reproducibility	Repeatability
Mean OD	198.4%	187.8%
SD	0.245	0.115
CV (%)	12.4%	6.1%

## Data Availability

The data supporting the findings of this study are available within the article. Additional data and information related to the study are available from the corresponding author upon reasonable request.

## Supplementary Materials

The following supporting information can be downloaded at: https://www.mdpi.com/article/10.3390/pathogens15060637/s1, Figure S1: Age-specific distribution of seropositive and seronegative animals; Figure S2. Species-specific distribution of seropositive and seronegative animals; Table S1: Performants’ assessment of the ELISA assay: repeatability and reproducibility (CV%);Table S2: Comparison of model fit statistics between the BLME and GLM models; Table S3. Sensitivity comparison between Bayesian mixed-effects and standard logistic regression models; Table S4. Pairwise comparisons among age groups and species; Table S5. BLME questionnaire.

## Abbreviations

The following abbreviations are used in this manuscript:

CCHFV	Crimean–Congo hemorrhagic fever virus
ELISA	Enzyme-linked immunosorbent assay
BLME	Bayesian logistic mixed-effects
