# Supplementary material for: Crimean-Congo Hemorrhagic Fever Infection in the Small Ruminant Population in N. Macedonia: A Seroepidemiological Study as a Step Towards Better Understanding of the CCHF Epidemiology in the Country
Source: Pathogens. 2026 Jun 16;15(6):637. doi: 10.3390/pathogens15060637 (PMC13304970; doi:10.3390/pathogens15060637)
Supplement: Supplementary file 1 [file pathogens-15-00637-s001.zip › pathogens-4357396-supplementary.pdf]

pathogens

Supplementary Materials

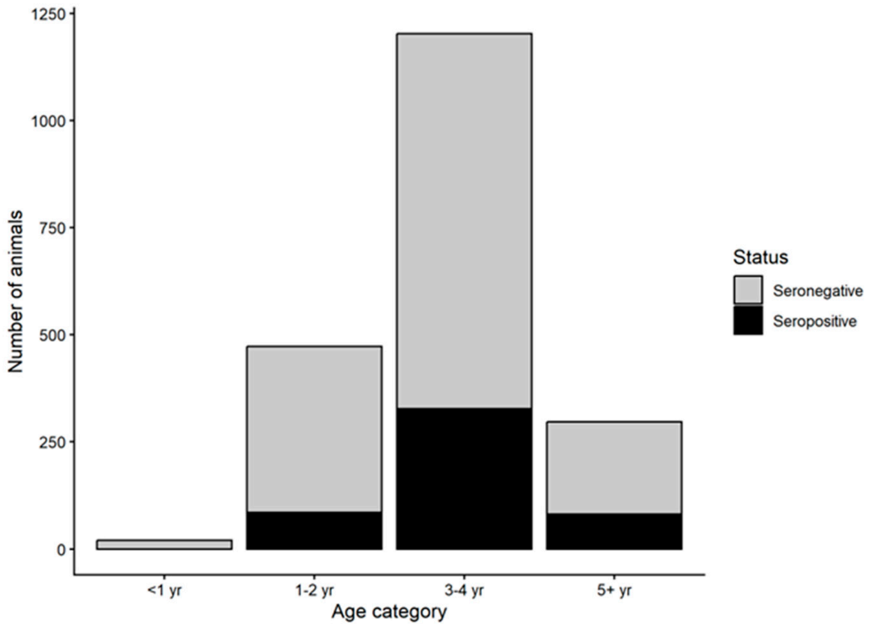

Figure S1. Age-specific distribution of seropositive and seronegative animals.

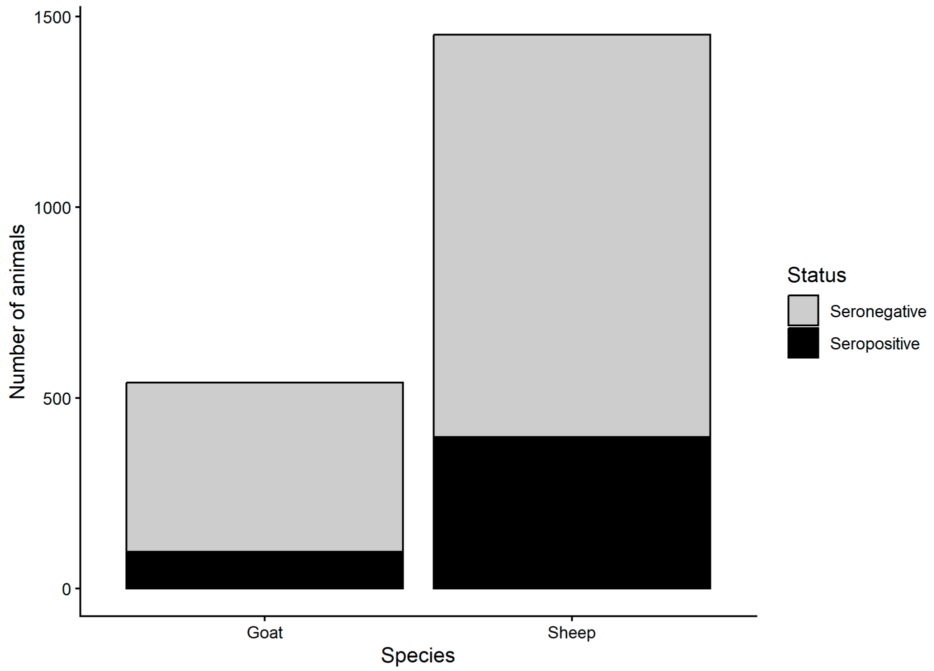

Figure S2. Species-specific distribution of seropositive and seronegative animals.

Table S1. Performants' assessment of the ELISA assay: repeatability and reproducibility (CV%)

	Reproducibility				Repeatability			
Replicate No	ELISA plate	Sample	OD	PP	ELISA plate	Sample	OD	PP
1	6	15810	1.706	191.4%	22	22960	1.914	210.1%
2	7	15810	1.540	180.0%	22	22960	1.837	201.6%
3	8	15810	1.787	191.5%	22	22960	1.661	182.3%
4	9	15810	1.847	189.7%	22	22960	1.730	189.9%
5	10	15810	1.902	216.3%	22	22960	1.809	198.6%
6	11	15810	1.768	248.0%	22	22960	1.690	185.5%
7	12	15810	1.507	178.6%	22	22960	1.554	170.6%
8	13	15810	1.697	185.9%	22	22960	1.592	174.8%
9	14	15810	1.620	194.8%	22	22960	1.792	196.7%
10	15	15810	2.018	232.8%	22	22960	1.626	178.5%
11	16	15810	2.357	225.9%	22	22960	1.626	178.5%
12	17	15810	1.644	177.3%	22	22960	1.679	184.3%
13	18	15810	1.772	166.8%	22	22960	1.737	190.7%

Table S2. Comparison of model fit statistics between the BLME and GLM models.

Model	AIC	BIC	LogLik
BLME	1184.66	1257.42	-579.33
GLM	1807.98	1875.14	-891.99

Table S3. Sensitivity comparison between Bayesian mixed-effects and standard logistic regression models.

Variable	OR BLME	BLME 95% CI	OR GLM	GLM 95% CI
Age<1 yr	0.00	0.00-58.00	0.00	0.00-49.00
Age≥5 yr	5.52	2.69-11.33	2.31	1.56-3.42
Age3-4 yr	3.08	1.84-5.16	2.02	1.51-2.69
Eastern	974.88	75.00-12671.87	54.18	23.01-127.56
Northeastern	28.21	2.39-332.41	22.25	9.40-52.68
Pelagonia	4.40	0.35-54.69	4.99	2.00-12.42
Southeastern	38.96	3.27-463.64	19.65	8.18-47.17
Skopje	137.92	10.09-1884.75	27.93	11.79-66.16
Southwestern	1.04	0.06-16.99	1.56	0.53-4.58
Vardar	1242.07	88.99-17337.13	44.25	18.79-104.21
Sheep	4.43	1.80-10.86	2.50	1.90-3.28

Table S4. Pairwise comparisons among age groups and species.

Contrast	Estimate	SE	z.ratio	p-value
1-2 yr vs <1 yr	13.943	161.909	0.086	1.000
1-2 yr vs ≥5 yr	-1.708	0.367	-4.654	< 0.001
1-2 yr vs 3-4 yr	-1.124	0.263	-4.269	< 0.001
<1 yr vs ≥5 yr	-15.651	161.909	-0.097	1.000
<1 yr vs 3-4 yr	-15.067	161.909	-0.093	1.000
≥5 yr vs 3-4 yr	0.584	0.306	1.910	0.224
Goat vs Sheep	-1.487	0.458	-3.247	0.001

*Statistically significant difference at $p \leq 0.05$, SE = standard error; z-ratio = Wald test statistic.

Table S5. BLME questionnaire

Variable	OR	95% CI	p-value
Terrain Lowland	0.15	0.00-4.82	0.285
Terrain Mountainous	0.07	0.00-4.24	0.205
Herd Size Medium	0.95	0.02-39.84	0.979
Herd Size Small	2.30	0.05-116.32	0.678
Herd Mixing Yes	0.08	0.00-70.15	0.459
New Animals Yes	0.03	0.00-4.42	0.171
Movement Range No Movement	0.00	0.00-Inf	0.971
Movement Range Outside Municipality	5393.99	0.93-31.70	0.052
Movement Range Within Municipality	0.80	0.03-23.47	0.898
Tick Exposure Low	0.00	0.00-6.97	0.147
Tick Exposure Moderate	0.02	0.00-57.12	0.350
Tick Exposure No Ticks	0.00	0.00-1.48	0.062
Tick Control Yes	4.57	0.13-15.31	0.400
